# ﻿*Mallomonas imperatoria* (Chrysophyceae, Synurales)—a new taxon from the tropics

**DOI:** 10.3897/phytokeys.269.179287

**Published:** 2026-01-07

**Authors:** Evgeniy S. Gusev, Nikita A. Martynenko, Irina N. Sterlyagova, Raisa M. Khatsaeva, Hoan Tran, Boris D. Efeykin, Marina E. Ignatenko

**Affiliations:** 1 Severtsov Institute of Ecology and Evolution, Russian Academy of Sciences, Leninsky Prospect, 33, 119071, Moscow, Russia Severtsov Institute of Ecology and Evolution, Russian Academy of Sciences Moscow Russia; 2 Komarov Botanical Institute of the Russian Academy of Sciences, St. Professor Popov, 2, 197022, St. Petersburg, Russia Komarov Botanical Institute of the Russian Academy of Sciences St. Petersburg Russia; 3 Institute of Biology, Komi Scientific Center, Ural Branch, Russian Academy of Sciences, Kommunisticheskaya Street, 28, 167982, Syktyvkar, Russia Komi Scientific Center, Ural Branch, Russian Academy of Sciences Syktyvkar Russia; 4 Joint Vietnam-Russia Tropical Science and Technology Research Center, 63 Nguyen Van Huyen, Nghia Do, Cau Giay, Hanoi, 11307, Vietnam Joint Vietnam-Russia Tropical Science and Technology Research Center Hanoi Vietnam; 5 Institute for Cellular and Intracellular Symbiosis, Orenburg Federal Research Center, Ural Branch, Russian Academy of Sciences, Pionerskaya Street, 11, 460000, Orenburg, Russia Orenburg Federal Research Center, Ural Branch, Russian Academy of Sciences Orenburg Russia

**Keywords:** Electron microscopy, *
Mallomonas
*, molecular phylogeny, new species, scale ultrastructure, section *Quadratae*, Synurales

## Abstract

This study investigates the *Mallomonas adamas* species complex in a tropical region using electron microscopy and molecular phylogeny. Analysis of four algal cultures from Vietnam, supplemented by data from two cultures from Europe and one from Australia, all belonging to the *M. adamas* morphotype, revealed two distinct clades in phylogenetic trees based on SSU rDNA + *rbcL* cpDNA and ITS1–5.8S–ITS2 rDNA sequences. Based on these results, a new species, *Mallomonas imperatoria***sp. nov.**, is described. *Mallomonas imperatoria* was found in 22 localities across nine provinces in Vietnam and is also known from Malaysia. This species generally prefers acidic water bodies, including those that are humic-stained, with low mineralization. Thus, findings of this taxon are not very frequent, but it cannot be classified as rare in the tropical region.

## ﻿Introduction

The genus *Mallomonas* Perty comprises approximately 250 species that have been studied using modern methods, including electron microscopy and molecular techniques (Siver 2024). Among the 21 sections within the genus *Mallomonas*, the section Quadratae Momeu & L.S. Péterfi is particularly notable. Its representatives are characterized by thick, robust scales and are typically large-celled species (Kristiansen and Preisig 2007). The thick scales are presumed to mitigate the effects of ultraviolet radiation (Němcová et al. 2024). This section may represent one of the ancient lineages in the evolution of the genus. The body scales of species in this section usually have a rhomboidal form, yet they exhibit a diversity of structures that determine scale orientation on the cell surface. While in most *Mallomonas* species this is governed by the so-called V-rib, various analogues of this structure are observed within the section Quadratae (Gusev et al. 2017b, 2019b, 2025). The morphology of the terminal scales (both apical/anterior and caudal/posterior) is also variable. A special case is presented by *M. adamas* K. Harris & D.E. Bradley, which lacks bristles—a feature characteristic of only a few members of the genus (Kristiansen and Preisig 2007). Thus, the group shows various evolutionary trajectories in the development of traits related to scale arrangement on the cell surface.

Recently, the section has been expanded with new species. *Mallomonas bronchartiana* Compère has been transferred to this section, and recently described morphologically similar species, such as *M. pseudobronchartiana* E.S. Gusev, P.A. Siver & W. Shin, *M. velari* E.S. Gusev, P.A. Siver & W. Shin, and *M. gusakovii* E.S. Gusev, D.A. Kapustin, N.A. Martynenko, E.E. Guseva & M.S. Kulikovskiy, have been included (Gusev et al. 2017b, 2019b). A recent revision of the *M. splendens* complex using molecular data revealed the presence of morphologically similar, closely related species, leading to the description of two new species, *M. tyleri* E.S. Gusev, Shkurina, H. Tran & Martynenko and *M. croomei* E.S. Gusev, Shkurina, H. Tran & Martynenko, and a taxonomic revision of *M. arnhemensis* (Croome, Dürrschmidt & Tyler) E.S. Gusev (Gusev et al. 2025). This brings the total number of species within the section to seventeen taxa.

Further molecular studies of species in this section have provided new data on the genetic diversity of *M. adamas*. This paper presents the results of these investigations and describes a new species.

## ﻿Materials and methods

Water samples from four localities in two provinces in Vietnam were used for algal culture isolation (Table 1). The study was supplemented by the examination of preserved samples from seven provinces of Vietnam (Table 2). Additionally, water samples from four provinces were also studied using the metabarcoding approach (Table 2). Samples were taken during expeditions of the Joint Vietnam–Russia Tropical Science and Technology Research Center (the “Ecolan 3.2” project) in 2012–2020. Descriptions of the climatic and geographical features of the provinces are given in previously published papers (Gusev et al. 2017a, 2019a; Gusev and Martynenko 2022; Tran et al. 2022). In general, this area has a tropical monsoon climate with high annual precipitation, varying in timing and amount between provinces (Schmidt-Thomé et al. 2015). Planktonic samples were collected using a plankton net with a 20 μm mesh size for culture isolation. Water mineralization and temperature measurements were performed using a Hanna device (HI 9828, Hanna Instruments, Inc., Woonsocket, RI, USA). Algal cultures were isolated by E.S. Gusev in 2012 (VN814, VN 816, VN 823) and 2018 (E4/22). Cultures were perpetually transferred to the Collection of the Severtsov Institute of Ecology and Evolution, Russian Academy of Sciences (IEE RAS).

**Table 1.** List of studied cultures with information about localities in Vietnam and environmental parameters: Cond.—specific conductance (µS cm^−1^); T—temperature (°C); SSU + ITS and *rbcL*—GenBank accession numbers.

Culture	Locality	GPS	pH	Cond.	T	SSU+ITS	*rbc*L
**Khanh Hoa Provinc**e							
VN814	Sandpit 1 in Cam Ranh Peninsula	12°04.927'N, 109°10.977'E	5.5	53	34	PX698440	PX703656
VN816	Sandpit 2 in Cam Ranh Peninsula	12°4.803'N, 109°11.030'E	5.6	40	36	PX698441	PX703657
VN823	Sandpit 3 in Cam Ranh Peninsula	12°05.040'N, 109°10.935'E	5.5	54	33	PX698442	PX703658
**Dong Nai Province**							
E4/22	Old irrigation pond in Cat Tien National Park	11°24.406'N, 107°24.388'E	6.4	43	32	PX698443	PX703659

**Table 2.** Basic characteristics of the localities (samples for morphological analysis and metabarcoding): No.—number of a sample; GPS—coordinates; Cond.—specific conductance (µS cm^−1^); T—temperature (°C); Chl *a*—chlorophyll *a* (µg l^−1^); n/a—data not available; *—metabarcoding data.

No.	Locality	GPS	pH	Cond	T	Chl *a*
**Gia Lai Province**						
1	Sandpit	13°50.223'N, 109°16.496'E	5.8	76	33	13
2	Reservoir Ho Giang	14°34.464'N, 108°59.776'E	6.3	62	35	35
3	Unnamed lake	14°03.975'N, 109°13.026'E	6.5	83	33	7
**Khanh Hoa Province**						
4	Freshwater pool on the seashore in Cam Ranh Peninsula	12°2.216'N, 109°12.169'E	5.5	87	29	n/a
5	Sandpit 4 in Cam Ranh Peninsula	12°04.709'N, 109°11.378'E	6.5	59	35	n/a
6	Pond	12°37.726'N, 109°06.644'E	6.3	54	31	n/a
7	Reservoir Lien Sang	12°16.049'N, 108°49.373'E	7.5	100	29	23
**Lam Dong Province**						
8	Hồ Nam Phượng (reservoir)	11°33.546'N, 107°49.281'E	6.9	18	25	12
9	Da Kha Reservoir*	12°06.572'N, 108°34.792'E	7.8	21	25	5
**Dong Nai Province**						
10	Ta Lai Reservoir (2012)	11°23.267'N, 107°21.050'E	6.4	37	30	n/a
	Ta Lai Reservoir (2021)*	11°23.267'N, 107°21.050'E	6.7	7	28	n/a
11	Temporary waterbody in Cat Tien National Park	11°23.538'N, 107°22.187'E	6.3	45	25	n/a
12	Unnamed Reservoir*	11°06.986^'^N, 107°03.355^'^E	6.1	28	30	n/a
**Phu Quoc Island, Kien Giang Province**						
13	Duong Dong Reservoir	10°15.039'N, 104°1.279'E	5.7	6	31	n/a
**Dak Lak Province**						
14	Unnamed reservoir	12°56.464'N, 108°49.025'E	6.9	33	33	4
**Hue Province**						
15	Lake Bau Co	16°33.337'N, 107°24.054'E	6.8	33	37	41
**Danang**						
16	Vinh Trinh Reservoir*	15°48.287'N, 108°09.839'E	7.3	30	27	15
**Binh Phuoc Province**						
17	Thac Mo Reservoir*	11°47.854^'^N, 107°01.057^'^E	6.4	24	27	n/a
18	Unnamed Reservoir*	11°39.527^'^N, 107°04.144^'^E	6.2	19	25	n/a

The study of the ultrastructure of scales of the *Mallomonas* species was carried out using scanning electron microscopy (SEM) on a Tescan Mira3 microscope (Tescan Brno, s.r.o., Brno, Czech Republic) at the Joint Usage Center “Instrumental methods in ecology” at the IEE RAS and the Gagarin Center for the Identification and Support of Talented Children (Orenburgskaya oblast), and also using transmission electron microscopy (TEM) on a JEM-1011 transmission electron microscope in the Center of Electron Microscopy at the Papanin Institute for Biology of Inland Waters, RAS. An aliquot of a sample was applied to SEM stubs, dried at room temperature, and sputtered with gold using an ion-plasma sputtering system (Quorum Q150R ES Plus; Quorum Technologies Ltd., London, UK). For studies with the transmission electron microscope (TEM), formvar-coated grids (EMS FF200-Cu-50, Electron Microscopy Sciences, Hatfield, PA, USA) were used.

Monoclonal cultures were established by examination of micropipetted single cells under an inverted microscope. Non-axenic unialgal cultures were maintained in modified WC, DY-V, and Waris-H liquid media (McFadden and Melkonian 1986; Andersen 2005) at 22 °C, in a growth chamber with a 12:12 h light:dark photoperiod with light intensity of 50–100 µmol m^−2^ s^−1^. In total, four cultures were isolated from different parts of Vietnam. They were used for further phylogenetic analysis based on SSU rDNA + *rbcL* cpDNA and ITS1–5.8S–ITS2 rDNA datasets.

The total DNA of the monoclonal culture was extracted using InstaGeneTM Matrix according to the manufacturer’s protocol. Fragments of partial SSU rRNA (1717 bp) were amplified using the primer pairs 18S-F (Katana et al. 2001) and 18L (Hamby et al. 1988). For ITS1–5.8S–ITS2 rRNA (596 bp) fragments, the primer pair KN1 (Wee et al. 2001) and Chryso_ITSR (Škaloud et al. 2012) was used. Amplification of the *rbcL* cpDNA (654 bp) marker was performed using the primers rbcL_2F (Daugbjerg and Andersen 1997) and Synura_rbcLR (Gusev et al. 2018). Amplification of all studied fragments was carried out using the premade mix ScreenMix (Evrogen, Russia) for the polymerase chain reaction (PCR). The conditions of amplification for partial rDNA fragments were an initial denaturation of 5 min at 95 °C, followed by 35 cycles of denaturation at 94 °C for 30 s, annealing at 52 °C for 30 s, and extension at 72 °C for 40–90 s, with a final extension of 10 min at 72 °C. The conditions of amplification for the *rbcL* fragments were the same as for ribosomal fragments, except for the number of cycles (40) and the annealing temperature (48 °C). The resulting amplicons were visualized by horizontal agarose gel electrophoresis (1.5%) and stained with SYBR Safe (Life Technologies, Carlsbad, CA, USA). Purification of DNA fragments was performed with the ExoSAP-IT kit (Affymetrix, Santa Clara, CA, USA) according to the manufacturer’s protocol. All studied fragments were sequenced from both directions using forward and reverse PCR primers and the BigDye system (Applied Biosystems, Foster City, CA, USA), followed by electrophoresis using a Genetic Analyzer 3500 sequencer (Applied Biosystems, Foster City, CA, USA). Additionally, fragments of SSU rDNA were sequenced using the internal primers 18S-826F (Choi et al. 2013) and picoR2 (Belevich et al. 2015) to assemble and verify the resulting sequences. The obtained sequences were checked manually and assembled using MegaX (Kumar et al. 2018).

Newly determined sequences and GenBank sequences of 81 other *Mallomonas* cultures were included in the alignment. In addition, synurophycean *Synura americana* Kynclová & Škaloud in Škaloud et al., *S. mammillosa* E. Takahashi, and *Neotessella lapponica* (Skuja) B.Y. Jo, J.I. Kim, W. Shin, P. Škaloud & P.A. Siver were added to the dataset as outgroup taxa. The sequences were aligned using either the global SILVA alignment in SINA v1.2.11 (Pruesse et al. 2012) for SSU rDNA or MAFFT v7 with the auto strategy (Katoh et al. 2019) for *rbcL* cpDNA fragments. Two separate phylogenetic analyses were performed: one based on concatenated partial SSU rDNA + *rbcL* cpDNA fragments and the other based on ITS1–5.8S–ITS2 rDNA sequences. The resulting SSU rDNA + *rbcL* cpDNA dataset (2371 bp) was partitioned into different genetic regions, and the most appropriate substitution model for each partition was estimated separately using the Bayesian information criterion in jModelTest 2.1.10 (Darriba et al. 2012). The GTR + G + I model was selected as the best-fit model for the SSU rDNA. For each codon position of the protein-coding *rbcL* cpDNA gene, the best model was also tested. The Bayesian information criterion-based model selection procedure selected GTR + G + I for the first codon position, JC + I for the second codon position, and GTR + G for the third codon position. The ITS1–5.8S–ITS2 rDNA dataset (612 bp) was constructed using 11 cultures from section Quadratae Momeu & Péterfi and aligned in MAFFT v7 with the auto strategy. The HKY + I substitution model was selected by jModelTest 2.1.10 for this dataset. Bayesian inference analysis was conducted using MrBayes 3.2.5 (Ronquist and Huelsenbeck 2003). Three hot and one cold Markov chains were run for 15 × 10^6^ cycles in two independent runs, sampling every 100^th^ tree. Phylogenetic trees and posterior branching probabilities were obtained after discarding the first 25% of trees as burn-in. For the SSU rDNA + *rbcL* cpDNA and ITS1–5.8S–ITS2 rDNA datasets, maximum likelihood phylogenies were constructed using IQ-TREE2 (Chernomor et al. 2016; Minh et al. 2020) with the models and partitions described above. The Bayesian inference tree was used as the starting tree for the maximum likelihood analysis, and bootstrap analysis with 1,000 replicates was performed. Viewing and editing of all phylogenetic trees were carried out using FigTree (ver. 1.4.2) and Adobe Photoshop CC (19.0).

The methodology of metabarcoding studies is described in previously published papers (Patova et al. 2023; Gusev et al. 2024).

## ﻿Results

**Figure 1.**
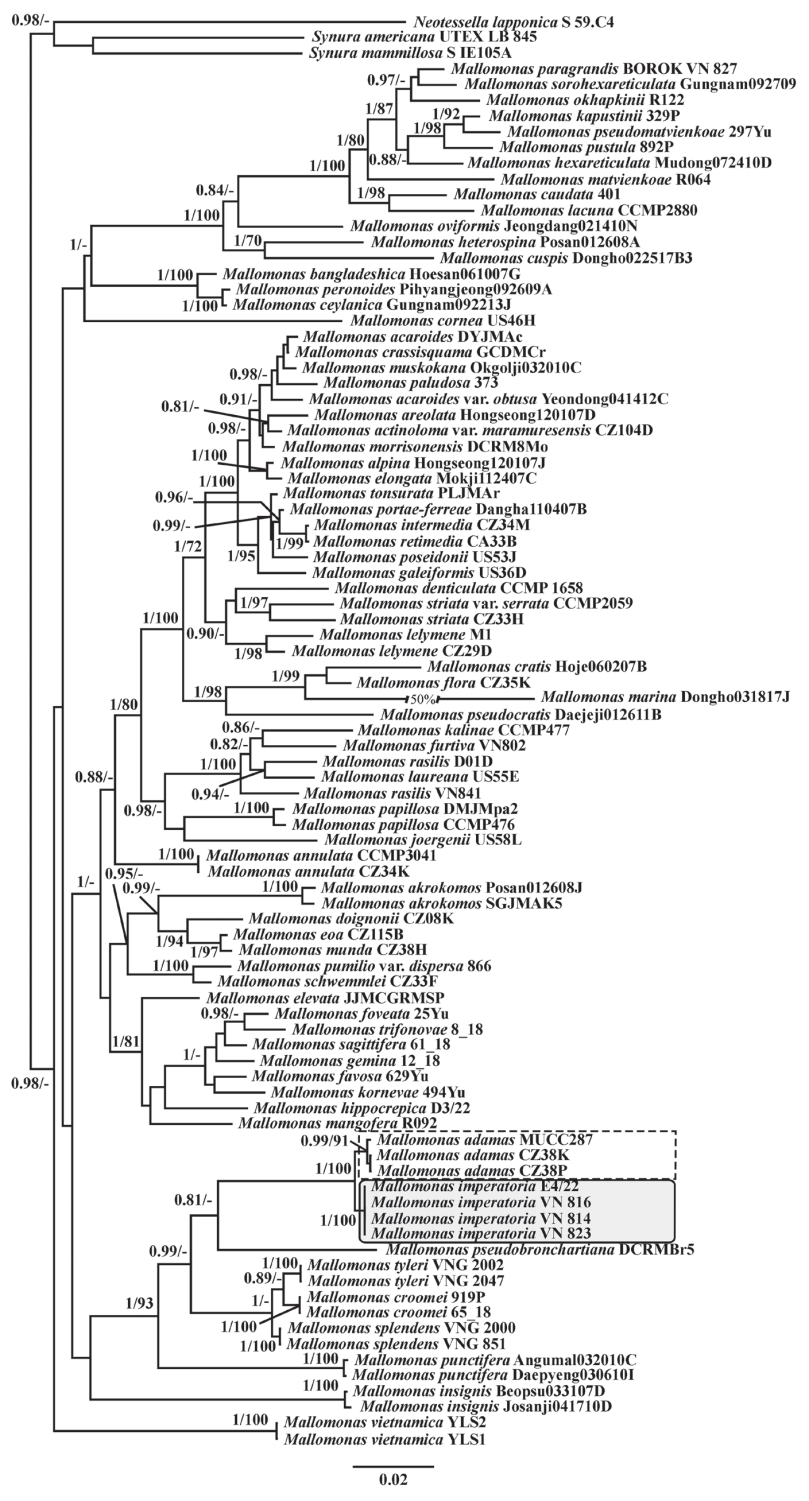
Bayesian consensus tree of the nuclear small subunit rDNA (SSU rDNA) and chloroplast *rbcL* concatenated dataset. The Bayesian posterior probability (> 0.80) and maximum likelihood bootstrap value (> 70%) are shown to the left and right of the fraction line, respectively. The scale bar represents substitutions per site. *Mallomonas imperatoria* sp. nov. is marked with a solid-line box, and *Mallomonas adamas* with a dashed-line rectangle.

**Figure 2.**
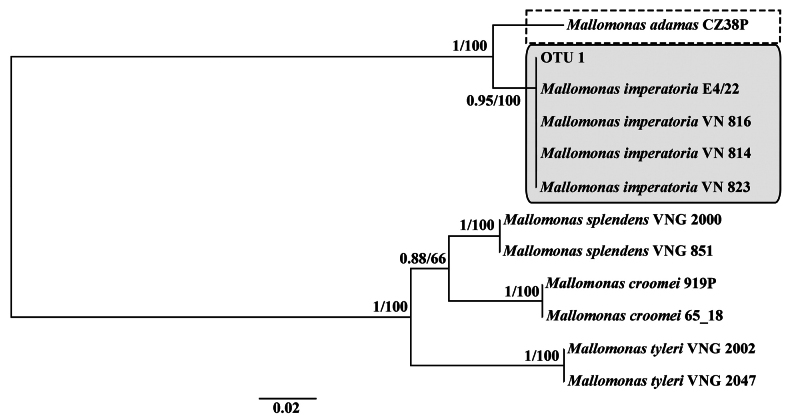
Unrooted Bayesian tree based on the ITS1–5.8S–ITS2 sequences of Mallomonas species from the section Quadratae. The Bayesian posterior probability and maximum likelihood bootstrap value are shown to the left and right of the fraction line, respectively. The scale bar represents substitutions per site. *Mallomonas imperatoria* sp. nov. is marked with a solid-line box, and *Mallomonas adamas* with a dashed-line rectangle.

In total, four algal cultures morphologically similar to *Mallomonas adamas* were isolated from four acidic water bodies during a study of freshwater chrysophytes in Vietnam (Table 1). Phylogenetic analyses based on concatenated datasets of nuclear-encoded SSU rDNA and plastid-encoded *rbcL*, as well as the ITS1–5.8S–ITS2 rDNA region, using Maximum Likelihood (ML) and Bayesian inference (BI), placed the cultures from Vietnam in a separate clade from the cultures CZ38K and CZ38P (Czech Republic) and MUCC287 (Australia), with high statistical support (Figs 1, 2). Distinctive morphological features of the Vietnamese organisms were also identified, including the complex structure of the papillae covering the scale surface and unique protrusions on the terminal scales. Based on these molecular and morphological differences, we describe a new species below.

### ﻿ Mallomonas imperatoria

Taxon classificationPlantaeSynuralesMallomonadaceae

E.S. Gusev, Martynenko, Sterlyagova & Ignatenko sp. nov.

1DF70EC9-D7A2-505C-A3F4-D9E959C790F8

Figs 3, 4, 5

#### Description.

Cells are elongated, cylindrical to ellipsoidal, 12.6–18.0 × 9.5–12.4 µm, covered with different scale types: body and terminal scales. All scales are thick, with an internal reticulation of closely spaced meshes and an external cover of papillae. Body scales are rhomboidal, slightly asymmetrical, 4.7–5.8 × 2.9–4.0 µm. The posterior rim is wide, encircling approximately half of the scale’s perimeter. The posterior flange is narrow. The shield is thick and raised above the flanges, covered with densely arranged papillae. The papillae are bipartite, consisting of a broad rounded base with a conical, short spine-like structure at the top. One side of the shield (the left) bears a developed rib, while the other side (the right) has a large, rounded depression. The part of the shield adjacent to this large depression lacks papillae. The anterior flange is narrow and smooth. The putative anterior scales are broadly ovate, asymmetrical, 4.6–5.0 × 3.2–3.4 µm, with a wide, rounded, or oval elevation in the distal part. This elevation is covered with papillae. On the shield, the papillae are displaced towards one edge. A large depression is present in the central part of the scale. The putative posterior scales are broadly ovate, 3.4–4.4 × 2.5–3.1 µm, with a prominent ridge of varying degrees of development located in the central part. Papillae cover the apical part of the ridge but are absent from the lateral walls. The scale surface surrounding the ridge is covered with papillae. Stomatocysts are unknown.

#### Type.

**Figure 3.**
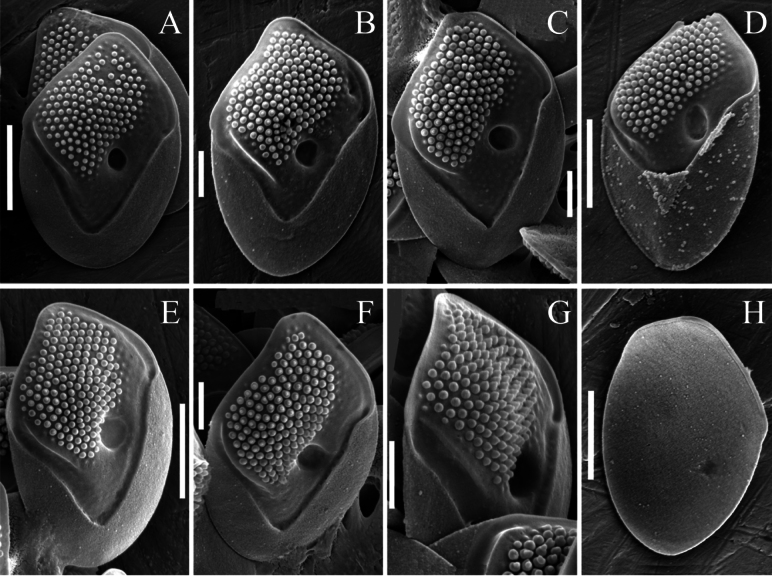
*Mallomonas imperatoria* sp. nov. (authentic culture VN814). **A–G.** Surface view of the body scales, SEM; **H.** Undersurface view of the body scale, SEM. Scale bars: 2 µm (**A, D, E, H**); 1 µm (**B, C, F, G**).

Vietnam • Sandpit, Cam Ranh Peninsula, Khanh Hoa Province; 12°04.927'N, 109°10.977'E; 25 June 2012; E.S. Gusev leg. Holotype. Portion of a single gathering of cells on SEM stub 74_I_1, deposited at the Herbarium of the Steppe Institute of the Ural Branch of the Russian Academy of Sciences, Orenburg (ORIS). Fig. 3С is a representative scale from the type specimen.

#### GenBank accession numbers for the reference culture (VN814).

PX698440 (nuclear SSU+ITS rDNA) and PX703656 (*rbc*L cpDNA).

#### Etymology.

The epithet “imperatoria” means “imperial”, referring to the distinctive crown-shaped ridge on the terminal scales.

#### Distribution and ecology.

In addition to the type locality, this species has been observed in 21 more localities in Vietnam (Tables 1, 2). *Mallomonas imperatoria* sp. nov. was found at quite wide ranges of environmental parameters: pH from 5.5 to 7.8, specific conductance from 6 to 100 µS cm^-1^, and temperature 25–37 °C, but it tends to prefer acidic conditions with low mineralized waters (Tables 1, 2).

**Figure 4.**
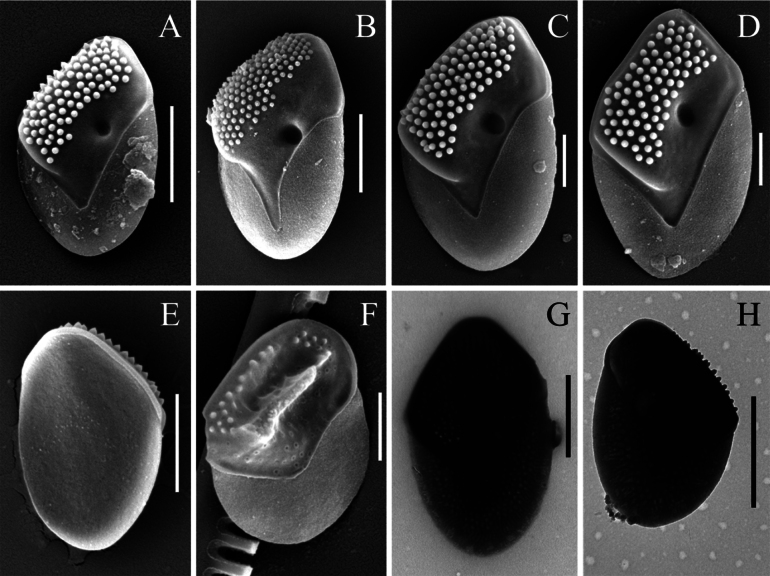
*Mallomonas imperatoria* sp. nov. (samples from Vietnam). **A–D.** Surface view of the body scales, SEM; **E.** Undersurface view of the body scale, SEM; **F.** Terminal scale with a developing ridge, SEM; **G, H.** Body scales, TEM. Scale bars: 2 µm (**A, B, E, G, H**); 1 µm (**C, D, F**).

**Figure 5.**
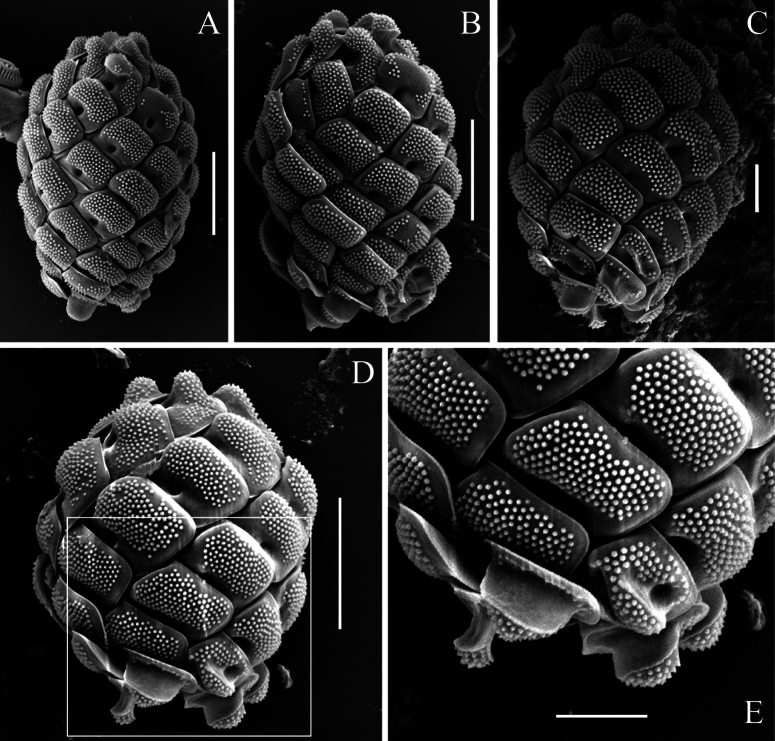
*Mallomonas imperatoria* sp. nov. (samples from Vietnam). **A–D.** Whole cells, SEM; **E.** Enlarged fragment of the posterior part of the cell with the terminal scales bearing a prominent ridge, SEM. Scale bars: 5 µm (**A, B, D**); 2 µm (**C, E**).

#### *Mallomonas imperatoria* sp. nov. (OTU1 nucleotide sequence, V9 SSU-ITS1 rDNA).

GCACCTACCGATTGAATGATTCGGTGAAAATTCCGGACTGTGGCTCGGTTGCTTTCGGGCGACTT TGCTGTAGGAAGTTATTTAAACCTCATCATTTAGAGGAAGGTGAAGTCGTAACAAGGTTTCCGTAG GTGAACCTGCGGAAGGATCATTACCACAACATCAACCACATTGATATCAACATATCATGCTGGTCC GATTAGCGCAATATAAAATTTAGTTTCTTCCGAAACTTTATTTTACTCCACAAGGATGTGGTATTC TGTTTGAGTGATTTTGGAGATCTCTAATCTCTCCAAACAAGGAGAGACATATGAAATTTCTAAACA CTTTCAGCAACGGATGTCTTGGCTCCCA.

## ﻿Discussion

*Mallomonas imperatoria* sp. nov. should be assigned to the section Quadratae based on its scale ultrastructure. Within this section, it is most similar in morphological structure and phylogenetic position to *M. adamas*. The species *M. adamas* was originally described from the United Kingdom (Harris and Bradley 1960). The authors noted that *M. adamas* was quite common in water bodies of peat bogs, forests, or gravel pits, where the water was not polluted by animal manure. They also observed that it appeared for short periods at any time of the year, but the specific conditions promoting its development remained unclear. Subsequently, this species has been recorded in several other countries: France (Němcová et al. 2012), the Czech Republic (Nováková et al. 2004; Němcová 2010), Australia (Dürrschmidt and Croome 1985), and Malaysia (Dürrschmidt and Croome 1985). In Vietnam, this morphotype was also found at 14 localities in various parts of the country (Gusev and Nguyen 2011; Gusev et al. 2017a, 2019a, 2022; Gusev and Martynenko 2022).

Data from molecular studies of *Mallomonas adamas* cultures isolated from water bodies in the Czech Republic (Němcová et al. 2024) and Australia (Lavau et al. 1997) have recently been published. In our study, cultures from Vietnam were isolated. This allowed for a comparison of different populations identified as *M. adamas*. The comparison revealed that the cultures from the Czech Republic and Australia, on one hand, and those from Vietnam, on the other, represent distinct genetic lineages. Although the genetic distance between the two species is relatively small, it corresponds to major differences in their morphology. The main ultrastructural differences lie in the structure of the papillae, the ultrastructure of the terminal scales, and slightly differing scale dimensions. We propose that these differences indicate the presence of two different species, which inhabit different climatic zones. A similar case was recently reported for two geographically distant species, *Mallomonas intermedia* Kisselev and *M. retimedia* Škaloud et al., which are almost indistinguishable morphologically (Škaloud et al. 2025).

The structure of the papillae differs significantly between the two species. The papillae of *Mallomonas adamas* can be described as simple, rounded structures, spaced approximately 2–3 times their diameter apart. Analysis of the available SEM images from temperate populations (Europe) revealed no complex composite structures. In contrast, the examined Vietnamese specimens possess larger papillae with a reduced inter-papillary distance, equal to or less than the papillae diameter (Fig. 3). They exhibit a more complex, bipartite structure—comprising a broad base and a narrower, conical, pointed apex mounted on top of it, which is clearly visible in SEM micrographs. This structure is consistent in both scales from cultures and those from fixed environmental samples, indicating that cultivation conditions do not alter papilla morphology. Furthermore, since the difference between European and tropical populations lies in the fundamental structure of the papillae, this character can be considered significant for taxon identification.

The second major distinction between *Mallomonas imperatoria* and *M. adamas* is the ultrastructure of the terminal scales. The terminal scales of *M. imperatoria*, both anterior and posterior, feature prominent ridges (elevations) of varying forms (Fig. 5). The putative anterior terminal scales possess a rounded elevation in the distal part, entirely covered with papillae. The putative posterior terminal scales bear a well-developed ridge, significantly raised in the central part of the shield. The ridge is covered with papillae on its top but has a smooth surface on its sides. In contrast, the terminal scales of *M. adamas* exhibit only slight elevations in the distal part of the anterior scales and small, narrow ridges (elevations) displaced toward the margin on the posterior scales (Lavau et al. 1997: fig. 1A, p. 138; Němcová et al. 2024: fig. 2f, p. 1259).

The scale dimensions of *Mallomonas adamas* and *M. imperatoria* also differ. For *M. adamas* from its type locality in the United Kingdom, scale sizes of 6–9 × 3.5–5 µm and cell sizes of 13–27 × 11–13 µm were reported (Harris and Bradley 1960). Calculations based on published scale images indicate that European populations have scales measuring 5.6–6.7 × 3.4–4.6 µm (Nováková et al. 2004; Němcová 2010; Němcová et al. 2012; Škaloud et al. 2013), with a mean size of 6.23 ± 0.06 × 4.14 ± 0.08 µm. Since the species was described from the UK and the Czech cultures also possess relatively large scales (ranging from 5.9 to 6.6 µm in length according to the provided images), we consider the Czech cultures to represent *M. adamas*.

In contrast, the scales of the *Mallomonas imperatoria* cultures from Vietnam are slightly smaller, measuring 4.7–5.8 × 2.9–3.9 µm, with a mean size of 5.42 ± 0.04 × 3.57 ± 0.03 µm. The scale sizes in the analyzed environmental samples varied within the range of 4.4–5.8 × 2.8–4.1 µm (Fig. 4). It must be mentioned that the maximum scale sizes in *M. imperatoria* overlap with the minimum sizes in *M. adamas*. Consequently, scale dimensions cannot be considered a reliable character for distinguishing these taxa. However, on the whole, scales from the tropical region are smaller. All scales published in studies on the flora of Vietnam have lengths of up to 5.6 µm. The published images from the tropics, referring to a record from Malaysia (Dürrschmidt and Croome 1985), should also be assigned to *M. imperatoria* based on their dimensions and the structure of terminal scales.

Among other representatives of the section Quadratae, *Mallomonas imperatoria* resembles the very rare species *M. parana* Vigna & Kristiansen, which is currently known only from Argentina (Vigna and Kristiansen 2005). The scales of both species are thick, with an internal reticulation and an external cover of papillae. The anterior scales in both species feature a rounded elevation. The structure of the papillae also appears to be similar, consisting of a base and a conical apex. However, there are significant differences between the species. Unlike *M. imperatoria*, the body scales of *M. parana* lack large rounded depressions. Furthermore, the body scales of *M. parana* are distinguished by the pattern of their internal reticulation, the internal structure of the posterior rim, and the presence of three small depressions (“windows”) in the angle of the V-rib. The anterior scales of *M. parana* possess two depressions, while in *M. imperatoria* there is only one. The putative posterior scales of *M. imperatoria* have a prominent ridge, whereas those of *M. parana* bear large spine-like papillae. The scales of the compared species also differ in size, being larger in *M. imperatoria*.

Due to the rhomboid and slightly asymmetric shape of its body scales, the internal reticulation with closely spaced meshes, the external cover of papillae, and its thick shield that is raised above the flanges, *Mallomonas imperatoria* shows some resemblance to the species *M. splendens*, *M. tyleri*, and *M. croomei* (Croome et al. 1985; Gusev et al. 2025). However, *M. imperatoria* is clearly distinguished by the structure of papillae, the presence of a rounded depression on the scales, the morphology of terminal cells, and the absence of bristles. *M. imperatoria* is also well separated from these species based on molecular data (Fig. 2).

*Mallomonas imperatoria* has been found in 22 localities across nine provinces in Vietnam. It was previously recorded in the country as *M. adamas* (Gusev et al. 2023). This species generally prefers acidic water bodies, including those that are humic-stained, with low values of specific conductivity. Thus, findings of this taxon are not very frequent, but it cannot be classified as rare in the tropical region.

## Supplementary Material

XML Treatment for Mallomonas imperatoria

## ﻿Additional information

### Use of AI

No use of AI was reported.

### Author ORCIDs

Evgeniy S. Gusev https://orcid.org/0000-0002-7397-5808

Nikita A. Martynenko https://orcid.org/0000-0002-7286-003X

Irina N. Sterlyagova https://orcid.org/0000-0003-2090-4114

Raisa M. Khatsaeva https://orcid.org/0000-0002-1009-9425

Hoan Tran https://orcid.org/0000-0002-0143-9918

Boris D. Efeykin https://orcid.org/0000-0003-1153-9995

Marina E. Ignatenko https://orcid.org/0000-0002-4451-7816

### Data availability

All of the data that support the findings of this study are available in the main text.
